# Single nucleotide polymorphism genes and mitochondrial DNA haplogroups as biomarkers for early prediction of knee osteoarthritis structural progressors: use of supervised machine learning classifiers

**DOI:** 10.1186/s12916-022-02491-1

**Published:** 2022-09-12

**Authors:** Hossein Bonakdari, Jean-Pierre Pelletier, Francisco J. Blanco, Ignacio Rego-Pérez, Alejandro Durán-Sotuela, Dawn Aitken, Graeme Jones, Flavia Cicuttini, Afshin Jamshidi, François Abram, Johanne Martel-Pelletier

**Affiliations:** 1 Osteoarthritis Research Unit, University of Montreal Hospital Research Centre (CRCHUM), 900 Saint-Denis, R11.412, Montreal, QC H2X 0A9 Canada; 2 Unidad de Genomica, Grupo de Investigación de Reumatología (GIR), Instituto de Investigación Biomédica de A Coruña (INIBIC), Complexo Hospitalario Universitario de A Coruña (CHUAC), Sergas, Universidade da Coruña, A Coruña, Spain; 3 Grupo de Investigación de Reumatología Y Salud (GIR-S), Departamento de Fisioterapia, Medicina Y Ciencias Biomédicas, Facultad de Fisioterapia, Universidade da Coruña, Campus de Oza, A Coruña, Spain; 4 Menzies Institute for Medical Research, University of Tasmania, Hobart, TAS Australia; 5 Department of Epidemiology and Preventive Medicine, Monash University, Melbourne, Australia; 6 Medical Imaging, ArthroLab Inc., Montreal, QC Canada

**Keywords:** Knee osteoarthritis, Prediction, Structural progressors, Early prognosis, Biomarkers, Machine learning, Single nucleotide polymorphism genes, mtDNA haplogroup

## Abstract

**Background:**

Knee osteoarthritis is the most prevalent chronic musculoskeletal debilitating disease. Current treatments are only symptomatic, and to improve this, we need a robust prediction model to stratify patients at an early stage according to the risk of joint structure disease progression. Some genetic factors, including single nucleotide polymorphism (SNP) genes and mitochondrial (mt)DNA haplogroups/clusters, have been linked to this disease. For the first time, we aim to determine, by using machine learning, whether some SNP genes and mtDNA haplogroups/clusters alone or combined could predict early knee osteoarthritis structural progressors.

**Methods:**

Participants (901) were first classified for the probability of being structural progressors. Genotyping included SNP genes *TP63*, *FTO*, *GNL3*, *DUS4L*, *GDF5*, *SUPT3H*, *MCF2L*, and *TGFA*; mtDNA haplogroups H, J, T, Uk, and others; and clusters HV, TJ, KU, and C-others. They were considered for prediction with major risk factors of osteoarthritis, namely, age and body mass index (BMI). Seven supervised machine learning methodologies were evaluated. The support vector machine was used to generate gender-based models. The best input combination was assessed using sensitivity and synergy analyses. Validation was performed using tenfold cross-validation and an external cohort (TASOAC).

**Results:**

From 277 models, two were defined. Both used age and BMI in addition for the first one of the SNP genes *TP63*, *DUS4L*, *GDF5*, and *FTO* with an accuracy of 85.0%; the second profits from the association of mtDNA haplogroups and SNP genes *FTO* and *SUPT3H* with 82.5% accuracy. The highest impact was associated with the haplogroup H, the presence of CT alleles for rs8044769 at *FTO*, and the absence of AA for rs10948172 at *SUPT3H*. Validation accuracy with the cross-validation (about 95%) and the external cohort (90.5%, 85.7%, respectively) was excellent for both models.

**Conclusions:**

This study introduces a novel source of decision support in precision medicine in which, for the first time, two models were developed consisting of (i) age, BMI, *TP63*, *DUS4L*, *GDF5*, and *FTO* and (ii) the optimum one as it has one less variable: age, BMI, mtDNA haplogroup, *FTO*, and *SUPT3H*. Such a framework is translational and would benefit patients at risk of structural progressive knee osteoarthritis.

**Supplementary Information:**

The online version contains supplementary material available at 10.1186/s12916-022-02491-1.

## Background

Osteoarthritis (OA), a debilitating musculoskeletal disease, is the main reason for permanent work incapacitation and seeing primary care physicians. The current therapies available to treat OA only relieve pain and not the structural alteration of the joint. Moreover, conventional diagnosis is ineffective in the early identification of patients in whom the disease will progress rapidly. This situation is a bottleneck for developing effective treatment aiming at the joint structure and attaining precision medicine. Finding biomarkers that will enable stratifying OA patients into subgroups/phenotypes will assist in a better understanding of individual patient needs and the development of disease-modifying OA drugs (DMOADs). In this line of thought, genetics have been shown to play an important role in the prevalence and progression of OA [1–5], and genetic markers are believed to be important for the stratification of patients with OA.

Extensive genome-wide association studies (GWAS) yielded several single nucleotide polymorphisms (SNPs) within different gene loci associated with OA and included *GDF5*, *MCF2L*, *TP63*, *FTO*, *DUS4L/COG5*, *GNL3*, *SUPT3H*, and *TGFA*, to name a few [1–10]. Some SNPs were specific for a population, joint site, and/or gender.

Mounting evidence also suggests the implication of some mitochondrial DNA (mtDNA) SNPs in the pathogenesis of OA. The mtDNA is exclusively maternally transmitted, and its sequence evolution rate is higher than the average nuclear DNA. As a result, a significant number of mtDNA mutations have accumulated sequentially along radiating maternal lineages [11]. These accumulated mtDNA mutations (haplogroups) are characterized by the presence of a particular set of SNPs in their sequence. The most frequent Caucasian mtDNA haplogroups are H, J, T, U, K, and others (the latter not ascribed to any of these haplogroups) [4, 12–15], in which the H variant was the most frequent (about 48%) [16]. In addition, haplogroups with a common phylogenetic origin are organized into clusters and named HV, TJ, KU, and C-others. Although these mutations have been critical for human adaptation, some may be maladaptive in a different environment with new lifestyles. This could have occurred as these mutations lead to modifications in cytoplasmic signaling molecules, thus reprogramming nuclear DNA gene expression [11, 17]. Moreover, certain are related to the pathogenesis of OA [4, 12, 18–23].

The need to develop tests to facilitate early and more appropriate therapeutic intervention is widely recognized and crucially required in the field of OA. The objective is to obtain not only an early and accurate diagnosis but also an early prognosis of the disease progression for a given individual. Precision and personalized medicine or at least stratified interventions could be achievable with biomarkers.

During the last few decades, researchers have looked at biomarkers mostly related to proteins in the serum/urine for an early diagnosis, monitoring, or prediction of the course of the disease. Yet, none is sufficiently specific or sensitive nor has been accepted by the regulatory agencies. In contrast to serum proteins, genes are not susceptible to daily activities and therefore are more stable. Having a genetic OA biomarker should provide a robust and powerful tool for the early identification of OA patients at risk of structural progression.

In recent years, instead of using individual features to identify progressors, a combination of OA markers and patient parameters conjointly with machine learning (ML) approaches have been found successful. However, studies using ML methodologies generally included a small number of patients, did not lead to robust predictions, and used radiography and/or symptoms to define OA progressors [24–31]. The two latter are well known to lack sensitivity to early knee structure (tissue) alterations and their changes over time [32, 33]. Moreover, symptoms are not recommended as, in addition to not correlating well with knee OA structural progression, they are very subjective and dependent on the population studied. However, combining radiography with quantitative magnetic resonance imaging (MRI) variables improves the identification of structural progressors [30]. Hence, MRI methodology is very sensitive to knee structural alterations, which could be detected even before morphological alterations are seen with other imaging-based technologies [34].

For the first time, the present study aims to determine, by using ML technologies, the gender-based predictability of some SNP genes and mtDNA haplogroups/clusters alone or combined with two OA major risk factors (age and body mass index [BMI]) in the risk of being a structural progressor of the knee. Knee structural progression was determined using features from both radiography and quantitative MRI. The developed models were validated using tenfold cross-validation experiments and an external OA cohort from the community-based Tasmanian Older Adult Cohort Study (TASOAC).

## Methods

### Study population

The DNA from the peripheral blood using buffy coat methodology was performed on 901 Caucasian (non-Hispanic white) individuals from the Osteoarthritis Initiative (OAI) baseline. In brief, the OAI established and maintained a natural history database for knee OA through yearly visits over 9 years that included 4796 participants divided into three subcohorts: control, incidence, and progressor. As previously described, selected participants were from the OAI progressor subcohort [20]. The name “progressor” of this subcohort was given based on having symptomatic and radiographic tibiofemoral knee OA at baseline. As our goal is to determine a predictability model in participants at risk of being a structural progressor of the knee, the participants were further classified as structural progressors or no-progressors (see below).

### Classification of structural progressors/no-progressors

Each participant (*n* = 901) was assigned a label for their probability values of being structural progressors (PVBSP), defined as progressors/no-progressors as previously described [35]. In brief, the classification was done using the developed ML algorithm in which the values of five features at baseline (two X-rays: joint space width [JSW] and joint space narrowing [JSN], and three MRI values: mean cartilage thickness of peripheral, medial, and central plateaus) were used as the input. Moreover, JSN ≥ 1 at 48 months was used as the outcome as in [35] it was ranked the most important outcome for discriminating structural progressors. Furthermore, a threshold of the prediction value was established for discriminating each participant into structural progressor/no-progressor. This was calculated with the $F1$ Max from the data model metrics as described [36], in which all predicted probabilities greater than or equal to the *F1* Max threshold are labeled progressors, and the *1-F1* Max threshold values are labeled no-progressors.

The OAI participant characteristics are shown in Table 1. Of the 901 individuals, 276 (31%) were labeled structural progressors and 625 (69%) no-progressors (Table 1).

**Table 1:** OAI participant baseline characteristics

	**Total** (*n* = 901)	**Progressors**^a^ (*n* = 276)	**No-progressors**^a^ (*n* = 625)	***p*-value**
Age, years	61 ± 9	63 ± 9	60 ± 9	**< 0.0001**
Gender, man, % (*n*)	39 (347)	41 (114)	37 (233)	0.266^b^
BMI, kg/m^2^	28.3 ± 4.6	28.9 ± 4.8	28.0 ± 4.5	**0.005**
WOMAC				
Pain (0–20)	2.1 ± 2.9	2.7 ± 3.4	1.9 ± 2.6	**0.0003**
Function (0–68)	6.8 ± 9.2	9.1 ± 11.1	5.8 ± 8.1	**< 0.0001**
Stiffness (0–8)	1.5 ± 1.5	1.7 ± 1.7	1.4 ± 1.4	**0.015**
Total (0–96)	10.4 ± 12.9	13.5 ± 15.6	9.0 ± 11.3	**< 0.0001**
Kellgren-Lawrence grade, % (*n*)				**< 0.0001**^b^
0–1	53 (476)	26 (73)	64 (403)	
2	34 (310)	41 (112)	32 (198)	
3	10 (93)	28 (77)	3 (16)	
4	2 (22)	5 (14)	1 (8)	
	(*n* = 796)	(*n* = 268)	(*n* = 528)	
Joint space width, mm	4.3 ± 1.3	3.2 ± 1.1	4.9 ± 1.0	** < 0.0001**
Joint space narrowing score^c^	0.4 ± 0.6	1.1 ± 0.5	0.1 ± 0.2	** < 0.0001**

*BMI* Body mass index, *WOMAC* Western Ontario and McMaster Universities Osteoarthritis Index, *mm* Medial minimum, *n* Number of participants

^a^Structural progressors and no-progressors were as defined in the “Methods” section

Continuous variables were compared using the Student’s *t*-test/Mann–Whitney test, and ^b^proportions using the chi-squared test/Fisher’s exact test; *p*-values compared progressors from the no-progressors, and a *p* ≤ 0.050 (in bold) was considered statistically different

^c^The joint space narrowing (JSN) scoring at baseline was 0–2, as described in the OAI database [37]

### Genotyping the predictors: SNP genes and mtDNA haplotypes/clusters

A set of eight SNPs, previously associated with the susceptibility to knee OA or with cartilage thickness in well-powered studies performed by robust GWAS, were selected [1, 3, 5, 10].

As previously described, the SNP mtDNA haplogroups were assigned using a single base extension (SBE) assay [18]. The haplogroups studied were the H, J, T, Uk, and others and the clusters HV, TJ, KU, and C-others. Of note, as the haplogroup K is a subtype of the U, these two haplogroups were combined as Uk.

The assignment approach of SNP genes was similar to that of the mtDNA haplogroups. In brief, a multiplex polymerase chain reaction (PCR) was performed to amplify the fragments that contain each of the informative SNPs in the following genes: rs12107036 at *TP63*, rs4730250 at *DUS4L*, rs10948172 at *SUPT3H*, rs11842874 at *MCF2L*, rs8044769 at *FTO*, rs11177 at *GNL3*, rs143383 at *GDF5*, and rs3771501 at *TGFA*. The resulting PCR fragments were further purified and assigned by the SBE assay, and the informative SNPs were visualized after loading the purified SBE product into an ABI 3130XL genetic analyzer (Applied Biosystems, Foster City, CA, USA). The assigned SNP genes and mtDNA haplogroups were verified by direct capillary sequencing in 30% of the samples.

### Model development

Twelve independent variables in PVBSP estimation were used and included the two major OA risk factors, age and BMI, and SNPs in eight genes, and mtDNA haplogroups/clusters as in Eq. 1.

1 $$\mathrm{PVBSP}=\mathrm{f}\left(\mathrm{age},\mathrm{ BMI},\mathrm{ mtDNA haplogroup},\mathrm{ cluster}, TP63, FTO, GNL3, DUS4L, GDF5, SUPT3H, MC2FL, TGFA\right)$$

A dominant model of the risk alleles was used to assess the influence of the SNPs as we assume that one copy of the risk allele is enough to modify the risk. The factors, age and BMI, were included as in addition to being interconnected with structural OA [38–40], some genetic polymorphisms, as well as mtDNA variants, have been found associated with these factors [41–47].

Additional file 1: Fig. S1 shows the frequency of eight SNP genes for the whole population, Additional file 2: Fig. S2 shows the association and frequency of mtDNA haplogroups and clusters in the study population (*n* = 901), and data are described in Additional file 3.

#### Gender-based model development for PVBSP forecasting

As it was reported that some genetic polymorphisms affect serum factors in a gender-dependent manner [47–51], we elected to build the models using gender separation, which would permit the highest accuracy. Consequently, the architecture of the ML methodologies could be different for each gender in that some parameters should be modified for each gender (Additional file 4), resulting in different models.

**Fig. 1:**
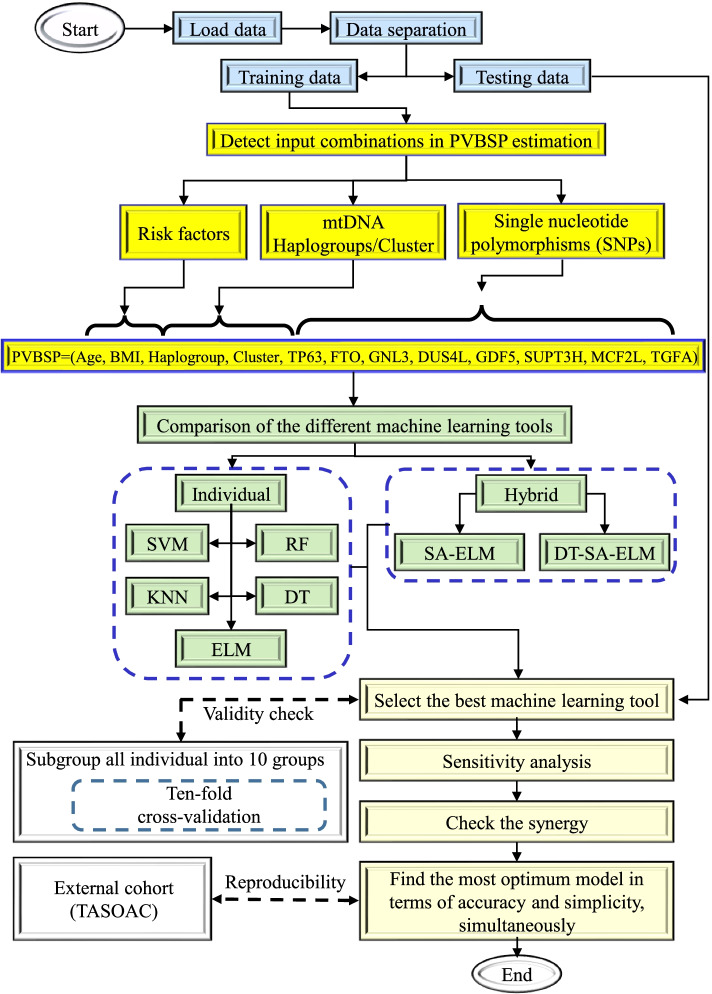
Flowchart of the proposed methodology in probability values of being structural progressors (PVBSP). DT, decision tree; DT-SA-ELM, decision tree and self-adaptive ELM; ELM, extreme learning machine; KNN, K-nearest neighbor; mtDNA, mitochondrial DNA; PVBSP, probability values of being structural progressors; RF, random forest; risk factors, age and bone mass index; SA-ELM, self-adaptive ELM; SVM, support vector machine; TASOAC, external cohort from the community-based Tasmanian Older Adult Cohort Study

Figure 1 illustrates the methodology used for PVBSP forecasting for each gender.

##### Data loading

The first step was data loading, which was randomly separated into training (70%) and testing (30%) datasets for both progressors and no-progressors.

##### Input variables

Next was the definition of the input variables in PVBSP forecasting consisting of the 12 above-described variables.

##### Machine learning models

For each gender, seven ML-based approaches (single and hybrid) were applied to model the PVBSP. These ML classification methodologies included the single algorithm support vector machine (SVM) [36], K-nearest neighbor (KNN) [52], random forest (RF) [53], decision tree (DT) [54], and extreme learning machine (ELM) [55], as well as the hybrid self-adaptive ELM (SA-ELM) [56], and a combination of decision tree and self-adaptive ELM (DT-SAELM). For details on their implementation, refer to Additional file 5.

##### Feature selection, sensitivity, and synergy analyses

As a convention, the model with a minimum of variables that estimates the outcome with high accuracy is desirable. We then analyzed if using lesser input variables can provide a simpler but accurate model compared to the one that uses all 12 variables. Using the best ML methodology found when the seven ML methodologies were compared, feature selection was then performed using a sensitivity analysis. To this end, all the 12 variables and the effect of each input variable were evaluated.

This was followed by evaluating the impact effect of the variable synergistically. For this analysis, two parameters were removed from the input variables and the accuracy of the new ML model was evaluated. This approach is directly linked to the model’s response with 12 variables with the synergy between two input variables for estimation of the PVBSP. Moreover, when one variable was linked to another, we defined it as a fixed variable, and the variable that could have an impact along with the fixed variable was considered a flexible variable. According to the accuracy of the models, three impact levels named highest, moderate and lowest were defined. Furthermore, we used the variables from the highest and moderate impacts and delineated two scenarios. The optimum model was selected from these analyses by simultaneously considering the accuracy and simplicity (i.e., lower number of input variables).

### Validation and reproducibility of the developed gender-based models

To ensure the generalization of the developed models, a two-step validation was performed. Internal validation was done using the tenfold cross-validation technique [57], and a reproducibility analysis was performed with an external cohort (TASOAC) [58] to check the generalizability of the models with a new data set.

Cross-validation was used to measure the skill of the established ML model in that it verifies how the results of the model may generalize to an independent dataset, removes the redundancy in the model ensuring its reliability with a different subgroup of the dataset, and prevents overfitting. The chosen model was evaluated using tenfold cross-validation, which measures the level of fitness in prediction to derive a reliable estimation of the model’s performance for independent datasets. Hence, all individuals were randomly and equally divided into ten different groups (also refer to Additional file 6: Fig. S3). One group was reserved as a test sample (validation), and the remaining nine were considered training samples. After training, the reserved sample (validation) was used to evaluate the model. This process was repeated ten times to check the accuracy of the ML models.

Each TASOAC participant was, as for the OAI, labeled for the PVBSP classification. From this cohort, 229 participants had the DNA and the required features for PVBSP classification. The TASOAC participant baseline characteristics are described in Table 2. Of the 229 individuals, 71 (31%) were labeled progressors and 158 (69%) non-progressors.

**Table 2:** TASOAC participant baseline characteristics

	**Total (*n* = 229)**	**Progressors**^a^ **(*n* = 71)**	**No-progressors**^a^ **(*n* = 158)**	***p*-value**
Age, years	62 ± 7	63 ± 7	61 ± 7	0.155
Gender, man, % (*n*)	48 (109)	37 (26)	53 (83)	**0.032**^b^
BMI, kg/m^2^	27.5 ± 4.5	28.3 ± 5.0	27.0 ± 4.1	0.127
WOMAC				
Pain (0–20)	1.3 ± 2.5	1.7 ± 2.8	1.2 ± 2.4	**0.045**
Function (0–68)	4.4 ± 9.0	5.6 ± 10.4	3.8 ± 8.3	***0.055***
Stiffness (0–8)	0.7 ± 1.3	1.0 ± 1.5	0.5 ± 1.2	**0.002**
Total (0–96)	6.4 ± 12.4	8.3 ± 14.0	5.5 ± 11.6	**0.027**
Joint space width, mm	4.7 ± 1.0	3.8 ± 0.8	5.1 ± 0.8	**< 0.0001**
Joint space narrowing, score^c^	0.5 ± 0.5	1.0 ± 0.2	0.3 ± 0.5	**< 0.0001**

*BMI* Body mass index, *WOMAC* Western Ontario and McMaster Universities Osteoarthritis Index, *mm* Medial minimum, *n* Number of participants

^a^Structural progressors and non-progressors were as defined in the “Methods” section

Kellgren-Lawrence was unavailable for the Tasmanian Older Adult Cohort Study (TASOAC) cohort. Continuous variables were compared using the Student’s *t*-test/Mann–Whitney test; ^b^proportions were compared using the chi-squared test/Fisher’s exact test; *p*-values compared progressors from the no-progressors, and a *p* ≤ 0.050 (in bold) were considered statistically different. The *p*-value in italic indicates that it did not quite reach the statistical significance

^c^The joint space narrowing (JSN) scoring was 0–2, as described [37]

## Results

### Participant characteristics

Comparison of the OAI cohort (Table 1) baseline characteristics between the structural progressors and no-progressors showed, for the former, a higher percentage of participants with a Kellgren-Lawrence (KL) score > 0–1, Western Ontario and McMaster Universities Osteoarthritis Index (WOMAC) scores and JSN, and a lower JSW. Age and BMI were also slightly higher in the structural progressor group, but although they reached statistical differences, they were not clinically significant.

For the TASOAC cohort (Table 2), a comparison between the two groups showed that the structural progressors have a higher WOMAC score and JSN, a lower JSW, and fewer men.

OAI and TASOAC cohorts have the same proportion for structural progressors (31%) and no-progressors (69%).

### Comparison of the machine learning methodologies

**Fig. 2:**
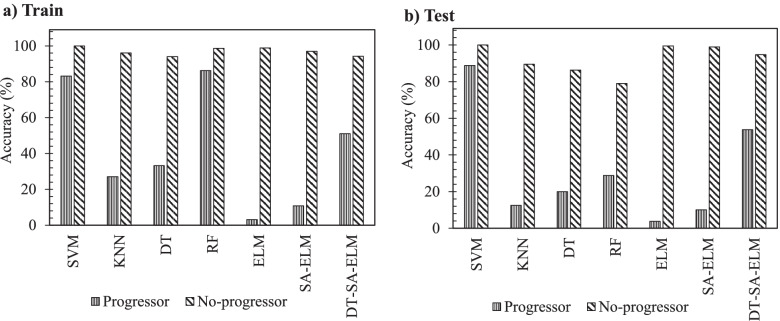
Comparison of the different machine learning methodologies in PVBSP in the OAI population. **a** Training (train) and **b** testing (test) stages accuracy for all the population. DT, decision tree; DT-SA-ELM, decision tree and self-adaptive ELM; ELM, extreme learning machine; KNN, K-nearest neighbor; OAI, Osteoarthritis Initiative; PVBSP, probability values of being structural progressors; RF, random Forest; SA-ELM, self-adaptive ELM; SVM, support vector machine; train, training stage; test, testing stage

With the OAI cohort, seven methodologies were compared using the 12 independent variables (Eq. 1). Figure 2 indicates the accuracy of the different ML methodologies in PVBSP forecasting at both the training and testing stages.

Data from the whole population showed that in the training stage (Fig. 2a), both the SVM and RF methodologies had good performances in all the population for structural progressors and no-progressors (mean of about 93%). The other methodologies resulted in poorer performances, primarily related to the progressor population. To select the superior model in PVBSP forecasting, the performance of different methodologies was analyzed in individuals who had not played a role in the calibration process, the testing stage (Fig. 2b). Data showed that only SVM demonstrated excellent accuracy for both groups. SVM methodology was thus further used for the development of the prediction models.

### Feature selection: sensitivity analysis

**Fig. 3:**
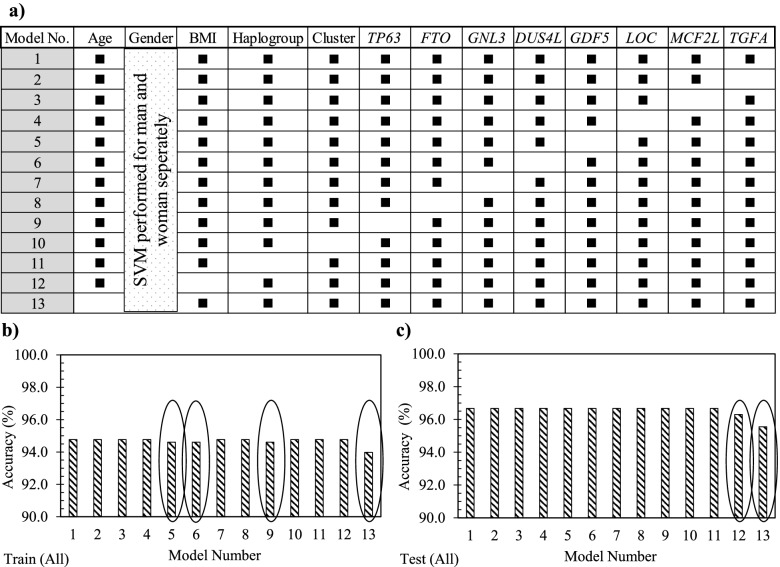
Sensitivity analysis using the support vector machine. **a** Representation of the different input combinations. Model number (No.) 1 includes all 12 variables, and one variable is removed in each of the others. A black rectangle indicates the used variable, while the white cells the non-used variable. In **b** and **c**, accuracy data in the training (train) and testing (test) stages of the developed support vector machine (SVM) with the different variable combinations using the whole (all) population (*n* = 901) is shown. The ovals in **b** and **c** indicate the models having a lower accuracy when the given variable is removed. BMI, body mass index

Using the whole OAI population, the effect of each 12 variables (Eq. 1) was evaluated in which all the models, except model 1, consisted of removing a variable (Fig. 3a). Data showed that model 1 had 94.8% accuracy in the training stage and 96.8% in the testing stage (Fig. 3b, c). Moreover, the removal of each variable demonstrated in the training stage (Fig. 3b) that not using *GDF5* (model 5), *DUS4L* (model 6), *TP63* (model 9), and age (model 13) as input variables slightly reduced the model’s accuracy in predicting PVBSP compared to model 1. At the testing stage (Fig. 3c), the variables BMI (model 12) and age (model 13) were also reduced compared to model 1. These data thus suggest that although the accuracy of these models is close, based on slight differences in training and testing stages, the important variables are age, BMI, *TP63*, *DUS4L*, and *GDF5*, and the following Eq. 2 can be considered as a model for PVBSP forecasting the progressor population:

2 $$\mathrm{PVBSP}=\mathrm{f}\left(\mathrm{age},\mathrm{ BMI}, TP63, DUS4L, GDF5\right)$$

When the population was divided into structural progressors and no-progressors, findings (data not shown) revealed that the difference found for the whole population was related to the progressor population. Hence, when only the progressor population was used, the model using all the 12 variables (model 1) showed an accuracy of 88.8% in the testing stage. For the no-progressors, there was no difference in the accuracy between the different models suggesting that the variables did not impact the outcome. Therefore, the no-progressor population was not detailed further.

### Machine learning model development

**Fig. 4:**
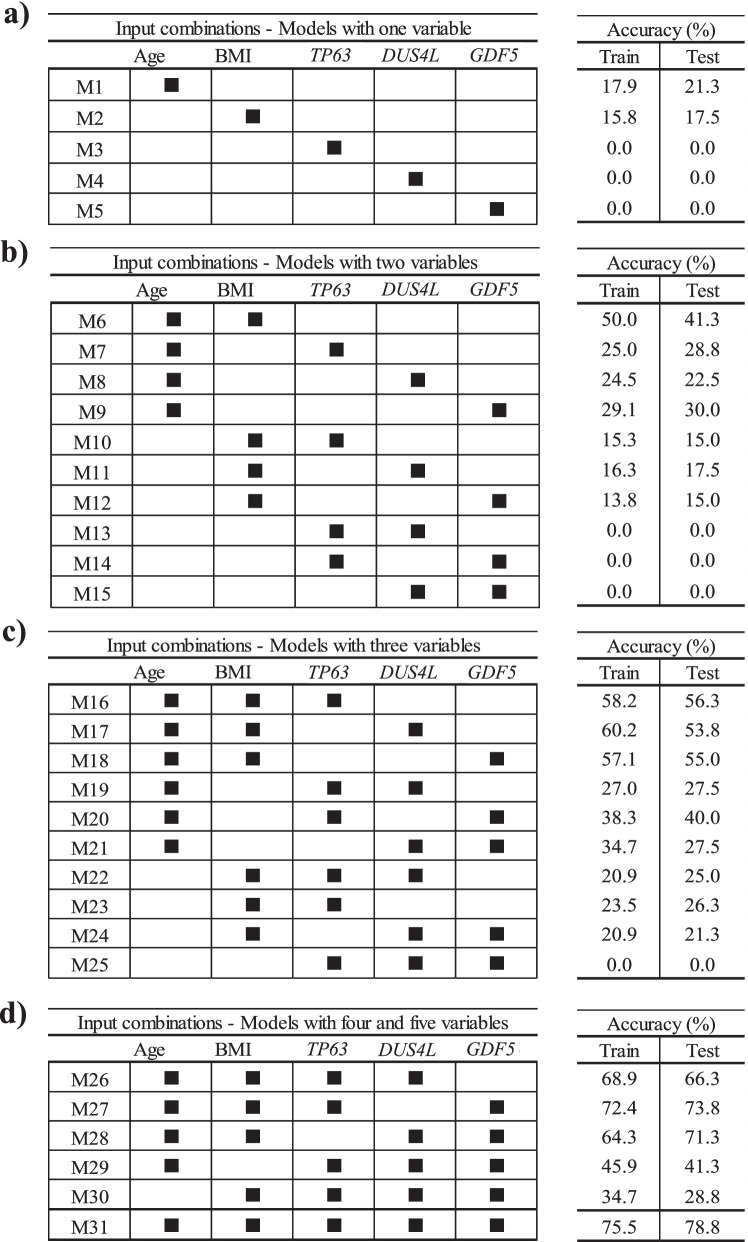
Finding the best input variable combinations. The combinations with the most important variables from sensitivity analysis for the progressor population used models built with **a** one variable, **b** two variables, **c** three variables, and **d** four and five variables. BMI, body mass index; M1–M31 number of models; train, training stage; test, testing stage

To find if lesser input variables can provide an accurate model, we further evaluated, by using the structural progressor population and the five variables as in Eq. 2, the scenarios of using one variable at a time followed by combining two to five variables. As illustrated in Fig. 4, 31 different ML models in PVBSP forecasting were developed. Data revealed that for models with only one variable (Fig. 4a), the accuracies of *TP63* (M3), *DUS4L* (M4), and *GDF5* (M5) at both training and testing stages were nil. Although the accuracy improved for age (M1) and BMI (M2), the numerical values (≤ 21.3%) were still very low. However, this improvement substantiates the importance of these two variables (age and BMI) as found in the sensitivity analysis (Fig. 3).

For the models with two variables (Fig. 4b), the highest accuracy (testing stage 41.3%) considered age and BMI simultaneously (M6). A comparison of the combination of each of these variables with age (M7–M9), with one consisting of age and BMI together (M6), showed that replacing BMI with *TP63* (M7), *DUS4L* (M8), and *GDF5* (M9) reduced the modeling accuracy in the testing stage by 12.5%, 18.8%, and 11.3%, respectively. However, if one of the inputs was BMI and the other two were one of the SNP genes (*TP63* [M10], *DUS4L* [M11], *GDF5* [M12]), the prediction accuracy was further reduced. Moreover, by not using age and BMI as one of the inputs of the models with two variables, we could not predict any of the progressors; the accuracy value was zero. These findings indicate that using *TP63*, *DUS4L*, and *GDF5* without the risk factors cannot yield an efficient model in PVBSP forecasting.

As shown in Fig. 4c, adding one of *TP63*, *DUS4L*, or *GDF5* as a variable to both age and BMI showed an increased accuracy. Models with three features that employed only one of the risk factors (M19–M21 for age and M22–M24 for BMI) had lower accuracy (range 21.3–40.0%) than the model with these two variables (M6, 41.3%). It should be noted that the simultaneous use of three variables without those of the two risk factors did not predict the progressors with high accuracy. Hence, the significant effect of age and BMI on PVBSP forecasting was confirmed.

For models with four and five variables (Fig. 4d), M26–M28 had higher accuracy than the best model offered among those using only three variables, i.e., M16. M27 demonstrated the best performance (testing stage, 73.8%). This model uses, in addition to age and BMI, *TP63* and *GDF5*. The combination of *DUS4L* and *GDF5* (M28) and *DUS4L* and *TP63* (M26) were in the second and third place, respectively. As for the models with fewer variables (Fig. 4b, c), the non-simultaneous use of BMI and age (M29 and M30) led to a model with lower accuracy.

These results (Fig. 4) show that increasing the number of inputs is effective when both age and BMI are considered. However, the best performance (testing stage 78.8%) was obtained with M31, which considers the five variables as in Eq. 2.

### Synergy of variables

The above data showed that, for the structural progressor population, the accuracy of M31 (five variables; testing stage, 78.8%) was lower than model 1 (12 variables; testing stage, 88.8%). Therefore, we assumed that some variables that are not considered in M31, including mtDNA haplogroup, cluster, *FTO*, *GNL3*, *SUPT3H*, *MCF2L*, and *TGFA*, could exert a synergistic effect with one or more variables in M31. This led to examining the synergy between the variables, and 66 new and different ML models were developed. Three impact levels were defined by comparing the results of each of these models with model 1 (Eq. 2) as the base model and according to the accuracy.

Table 3 illustrates, from highest to lowest, the impact effect between a fixed variable (variable 1) and one of the variables as listed in variable 2. Data revealed that the highest synergy impact was found for age with (i) BMI, (ii) *GNL3*, (iii) *MCF2L*, and (iv) *FTO*. Those having a moderate impact were age with (i) mtDNA haplogroup, (ii) *GDF5*, (iii) *SUPT3H*, (iv) *TGFA*, and (v) *TP63*; BMI with (vi) *TP63* and (vii) *SUPT3H*, and (viii) *GDF5* and *MC2FL*.

**Table 3:** Impact effect of the variable synergies in PVBSP forecasting

PVBSP forecasting	Variable 1	Variable 2
Highest impact	Age	BMI, *GNL3*, *MCF2L*, *FTO*
Moderate impact	Age	mtDNA haplogroup, *GDF5*, *SUPT3H*, *TGFA*, *TP63*
	BMI	*TP63*, *SUPT3H*
	GDF5	*MCF2L*
Lowest impact	TGFA	mtDNA haplogroup, cluster, *FTO*, *GNL3*, *SUPT3H*, *MCF2L*
	SUPT3H	mtDNA haplogroup, cluster, *FTO*, *GNL3*
	GNL3	mtDNA haplogroup, cluster, *FTO*
	FTO	mtDNA haplogroup, cluster
	Cluster	mtDNA haplogroup

Variable 1 indicates a fixed variable, and variable 2 is a flexible variable that could have an impact along with variable 1

*PVBSP* Probability values of being structural progressors

According to the highest and moderate impacts, two different scenarios were defined to find the optimum model (Table 4). In scenario 1, in addition to the combination of age and BMI, the factors found to have a high impact on synergies, *GNL3*, *MCF2L*, and *FTO*, were added one at a time to M31. In scenario 2, as the mtDNA haplogroup showed a moderate impact with age, it was used in addition to age and BMI as a fixed input variable, and the other SNP genes, *TP63*, *FTO*, *GNL3*, *DUS4L*, *GDF5*, *SUPT3H*, and *TGFA*, were added one at the time or in combination. All of the SNP genes were tested to ensure the accuracy and reliability of the final results.

**Table 4:** Synergy analysis in PVBSP forecasting

**Scenario 1**			
**Model**	**Input combinations**	**Accuracy (%)**	
		**Train**	**Test**
Model 1	Equation 1 (12 variables)	83.2	88.8
M31	Equation 2 (5 variables)	75.5	78.8
M32-1	M31 + *GNL3*	81.4	82.7
M32-2	M31 + *MCF2L*	77.0	81.3
M32-3	M31 + *FTO*	82.7	85.0
**Scenario 2**			
**No. of inputs**	**Model no**	**Accuracy (%)**	
		**Train**	**Test**
(3 + 1) variables	**MH2**	75.5	80.0
Age, BMI, mtDNA haplogroup, *FTO*			
(3 + 2) variables	**MH17**	85.2	82.5
Age, BMI, mtDNA haplogroup, *FTO*, *SUPT3H*			
(3 + 3) variables	**MH46**	82.1	88.8
Age, BMI, mtDNA haplogroup, *FTO*, *SUPT3H*, *GNL3*			
(3 + 4) variables	**MH80**	82.7	88.8
Age, BMI, mtDNA haplogroup, *TP63*, *DUS4L*, *GDF5*, *TGFA*			
(3 + 5) variables	**MH101**	83.2	88.8
Age, BMI, mtDNA haplogroup, *TP63*, *GNL3*, *DUS4L*, *GDF5*, *TGFA*			
(3 + 6) variables	**MH106**	83.2	88.8
Age, BMI, mtDNA haplogroup, *TP63*, *FTO*, *SUPT3H*, *GNL3*, *DUS4L*, *GDF5*			

Model 1 is Eq. 1: PVBSP = f(age, BMI, mtDNA haplogroup, cluster, *TP63*, *FTO*, *GNL3*, *DUS4L*, *GDF5*, *SUPT3H*, *MC2FL*, *TGFA*), and M31, Eq. 2: PVBSP = f(age, BMI, *TP63*, *DUS4L*, *GDF5*)

*M* Model, *No.* Number, *PVBSP* Probability values of being structural progressors, *test* Testing stage, *train* Training stage

#### Scenario 1

Three different models (Table 4, scenario 1) named M32-1 to M31-3 were defined and included the five variables of the model M31 plus *GNL3*, *MCF2L*, or *FTO*, respectively. The performance in the testing stage of M32-1 (82.7%), M32-2 (81.3%), and M32-3 (85.0%) was slightly lower than model 1 (12 variables, 88.8%) but higher than that of M31 (five variables, 78.8%). M32-3 (M31 + *FTO*) outperformed M31 and M31-2. Therefore, and to have a model with a lower number of variables, M32-3 appeared to be a very good model and consisted of:

3 $$\mathrm{PVBSP}=\mathrm{f}\left(\mathrm{age},\mathrm{ BMI}, TP63, DUS4L, GDF5, FTO\right).$$

#### Scenario 2

To verify if we could obtain a better accuracy with fewer variables, we analyzed another scenario consisting of age, BMI, and mtDNA haplogroup as fixed variables with one to seven SNP genes. This resulted in 109 combinations with four to ten variables and was named MH1-109. The analyses of all input combinations showed that the best accuracy range at the testing stage was 80.0–88.8%, and only those are represented in Table 4, scenario 2. Data showed that for four models with six to nine variables, the accuracy was identical (MH46, MH80, MH101, and MH106) in the testing stage (88.8%) as the one for model 1 with 12 variables. The model MH2 with four variables was at 80.0%, and MH17 with five variables at 82.5%. Given that the optimum model should not only have an excellent accuracy but the least number of variables, MH17 was selected as the optimal model:

4 $$\mathrm{PVBSP}=\mathrm{f}\left(\mathrm{age},\mathrm{ BMI},\mathrm{ mtDNA haplogroup}, FTO, SUPT3H\right).$$

### Effect of each variable on the optimum model, MH17

**Fig. 5:**
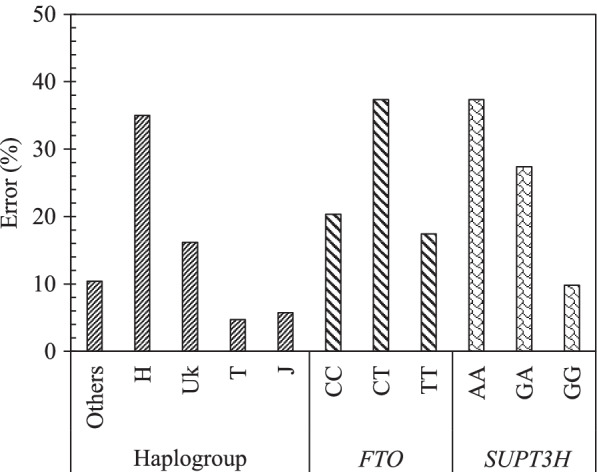
Effect of each variable of the model MH17. Impact of each mtDNA haplogroup and genotype alleles on the accuracy of the model MH17: PVBSP = f(age, BMI, mtDNA haplogroup, *FTO*, *SUPT3H*) for the progressor population. The high percentage of error indicates the highest impact of the variable. rs8044769 at *FTO*, presence of CC and CT, absence of TT: the risk allele C; rs10948172 at *SUPT3H* absence of AA, presence of GA and GG

The effect of each variable on the model MH17 was done using sensitivity analysis. Figure 5 demonstrates the impact of each SNP mtDNA haplogroup (others, H, Uk, T, J) and gene genotype for *FTO* (CC, CT, and TT) and *SUPT3H* (AA, GA, and GG) in PVBSP forecasting. The high percentage of error indicates the high impact of the studied variable.

Data showed that the mtDNA haplogroup H has the highest impact with an error of 35.0%, followed by UK with 16.1%, and ≤ 10% for the mtDNA haplogroup others, T, and J. FTO and SUPT3H both showed an identical highest error (37.3%) for both the presence of CT and absence of AA, respectively. The lowest error of *FTO* and *SUPT3H* was attained for the absence of TT (17.4%) and the presence of GG (9.8%), respectively.

### Validation of the developed models using cross-validation and reproducibility with an external cohort (TASOAC)

The performance of M32-3 (Eq. 3) and MH-17 (Eq. 4) models when using the ten repetitions of tenfold cross-validation showed an average accuracy of 95.1% ± 2.1 for M32-3 and 94.6% ± 2.1 for MH-17 (Additional file 7: Fig. S4).

Reproducibility experiment with the external cohort TASOAC also demonstrated an excellent accuracy for both M32-3 (90.5%) and MH17 (85.7%), confirming the reliability and performance of these two developed ML models in the early detection of at-risk knee OA structural progressors.

## Discussion

The current study’s main goal was to improve the clinical prognosis of knee OA for a better therapeutic approach. In this perspective, genetic biomarkers hold great potential for improving clinical outcomes in OA. We investigated, using ML methodologies, the prediction of SNP genes and mtDNA haplogroups/clusters as early predictors of knee OA structural progression.

All the SNPs evaluated in this work were shown to be related to OA [1, 3, 5–10]. Likewise, the mtDNA haplogroups/clusters H, J, T, Uk, and others and the clusters HV, TJ, and KU have all been associated with the disease. Hence, the J and T, as well as the cluster TJ, have not only been associated with a decreased risk of knee OA but also with a lower rate of incidence and progression of knee OA [4, 12, 18–21, 23] and that patients belonging to cluster TJ had a slower OA progression than patients belonging to cluster KU [22]. In contrast, patients with knee OA carrying (i) the haplogroup U and the cluster KU showed a more severe progression of the disease [18], (ii) the haplogroup H was more prone to OA progression and also total joint replacement [12, 19, 22], and (iii) the cluster HV had a marginal correlation with OA [59].

Data-driven approaches were used as such methodologies do not require an a priori hypothesis and are, therefore, able to identify unanticipated patterns in the data and offer the potential to provide new insights. These methods are widely used in medical research but only recently applied to OA. In an ML-based study, one of the important challenges is the selection of an appropriate supervised methodology enabling optimal performance in classifying the dataset. Here, we compared seven ML techniques in which each of them was fine-tuned with hyperparameters. Data showed that the supervised SVM methodology had the highest accuracy.

Using the SVM ML methodology, the gender-based models consisting of all the variables (*n* = 12) had high accuracy (88.8%). However, in general, a model should have a minimum of features in addition to the maximum possible accuracy to be more easily applicable. To this end, and to determine the optimum model, 277 models were evaluated, and two gender-based scenarios were developed. The first ones (scenario 1) consisted of age, BMI, and four SNP genes (*TP63*, *DUS4L*, *GDF5*, *FTO*) with an accuracy of 85.0%.

Furthermore, a second scenario was developed by exploiting data from the synergy analysis, in which a moderate level of synergy is found between age and mtDNA haplogroup, and using them as a fixed variable in addition to the BMI. This latter consisted of one less variable and included the three fixed ones (age, BMI, and mtDNA haplogroup) in addition to the SNP genes, *FTO* and *SUPT3H*, and with excellent accuracy (82.5%). Therefore, the latter was selected as the optimum model to predict early structural OA knee progressors, as it has one less variable than the other model. In this model, the mtDNA haplogroup H, as well as the presence of the alleles CT for rs8044769 at *FTO* and the absence of AA for rs10948172 at *SUPT3H*, demonstrated the highest impact.

The involvement of the mtDNA haplogroup H as a predictor of poor OA prognosis is not new. Different studies demonstrated that compared to other mtDNA haplogroups, especially those belonging to the mtDNA cluster JT, patients with the haplogroup H (or cluster HV) show a higher rate of OA incidence and progression [4, 12]. Among the proposed functional consequences of harboring this variant, higher free radical production, lower cell survival under oxidative stress conditions, and a higher grade of apoptosis stand out [12].

In addition, the effect of specific nuclear risk alleles can be conditioned by the mitochondrial background and vice versa through mitonuclear interactions [60–62]. This was reported in diseases such as Alzheimer’s, where an association between the cluster HV and the risk of this disease following adjustment for the *apolipoprotein E* gene (APOE4) status was detected [63], and in obese patients with type 1 diabetes mellitus [45]. Mitonuclear interactions have also been described in terms of the differential association of the haplogroups H and J with the methylation status of articular cartilage by which apoptosis, among other biological processes, is enhanced in cartilage with the haplogroup H and repressed in those having the haplogroup J [64].

Interestingly, the rs8044769 at the *FTO* variant was found to be linked to OA via its effect on the BMI [43]. Taking into account, on the one hand, previous associations of mitochondrial background with the risk of obesity [44–46] and, on the other hand, the differential methylation pattern between haplogroups H and J in cartilage in genes related to developmental processes, including the homeobox family [64], potential interactions between haplogroup H/cluster HV able to modify the risk of structural progression in OA are not surprising.

This study has several strengths. The population included a sensible number of participants for both genders, enabling the models to be developed in a gender-based fashion, permitting a high accuracy of the models. The validation and reproducibility of the developed models using cross-validation and an external cohort, respectively, demonstrated excellent accuracies for both M32-3 and MH17 models, reinforcing the robustness and generalizability of the developed models. In addition, OAI and TASOAC cohorts consist of people in the mild-moderate stage of the disease, thus representing a general population. Another strength is that, for classifying joint structural progressors (PVBSP) for each individual, and as suggested by Nelson et al. [30], we applied an image-based prediction algorithm using both radiographic and MRI variables and an overtime X-ray as the outcome from our previous study [35]. Finally, the development of our models using genetic and demographic information could have improved the ability of the models to predict knee structural alterations compared to having only genetic information, as previously described [65]. Moreover, incorporating modifiable risk factors (e.g., BMI) could also have increased the accuracy of the predicted models, such as previously described for knee OA [66].

Like all studies, the present has limitations. First, the participants were all of Caucasian origin; therefore, the results of this study did not extend beyond this ethnicity. Second, although we used the most common SNP genes and Caucasian mtDNA haplogroups associated with OA, others could also be tested. Third, for some SNP genes, the number of participants having a specific allele was limited (Additional file 1: Fig. S1). Fourth, although unbiased evaluation of models in training and testing stages were confirmed by tenfold cross-validation, one might argue that the results from the OAI cohort be optimistic in forecasting PVBSP as a nested cross-validation could have been used. However, this concern is alleviated by the validation analysis using an independent external cohort in which an excellent accuracy was obtained for both developed ML models. Fifth, we acknowledge that one of the important challenges in performing this study was the proximity of the results in the development of the models, and more specifically, in the sensitivity analysis. Hence, the results of the different models for SNP genes and mtDNA haplogroups/clusters were very close, and the important variables were selected based on small differences using both training and testing stages. However, by doing this, we were able to decrease the number of variables from 12 to only five or six, while maintaining high accuracies for the models.

Results from this study are translational for Caucasians at risk of structural progressive knee OA and could have high and direct clinical relevance as they could improve clinical prognosis with real-time patient monitoring. The next step will be to transform these automated models of OA knee structural progressors into an application that will make it practical for use by clinicians for a given patient. These models could be used early during the OA process and guide clinicians to adapt the therapeutic strategy to improve long-term harmful outcomes. In addition, they could assist in the design of knee DMOAD clinical trials. As the disease progression may be slow for many OA patients, DMOAD trials require extremely large numbers of patients and longer follow-up periods. However, stratifying patients who will likely have more rapid knee structural progression would result in enriched trials with appropriate patients as a result of discriminating potential responders from non-responders for a given therapeutic approach. Such a selection of OA patients, which at present is a major hurdle for DMOAD clinical trials, would result in lower trial costs, opportunities for testing more products, and faster end results.

## Conclusions

Understanding the links between efficient therapies and customized treatment plans in OA requires the ability to subgroup the patients at an early stage of the disease. ML provides reliable methodologies to classify these patients for tailoring decisions/treatments to individuals and to improve the recruitment of the right patient for a clinical trial. This study introduces a novel source of decision support in precision medicine in which, for the first time, two models consisting of (i) age, BMI, *TP63*, *DUS4L*, *GDF5*, and *FTO* and (ii) age, BMI, mtDNA haplogroup, *FTO*, and *SUPT3H* with the latter having one less variable are considered the optimum one. Such a framework would benefit Caucasian patients at risk of structural progressive knee OA, as it will personalize and improve the care of patients with knee OA.

## Supplementary Information

**Additional file 1: Fig. S1.** Frequency of single nucleotide polymorphism (SNP) genes in the studied population (n=901). We performed a dominant model of the risk alleles for the 8 SNPs: rs12107036.*TP63*, rs4730250. *DUS4L*, rs10948172.*SUPT3H*, rs11842874.*MCF2L*, rs8044769.*FTO*, rs11177.*GNL3*, rs143383.*GDF5*, rs3771501.*TGFA*. The column indicates the frequency for all the population. No, absence of the allele; NP, the number of no-progressors; P, the number of progressors; Yes, presence of the allele. Progressors and no-progressors are defined in the Methods section.

**Additional file 2: Fig. S2.** Association and frequency of mtDNA haplogroups and clusters in the studied population (n=901). **a**) Association between mtDNA haplogroups and clusters. The number of participants is indicated in parenthesis and above the arrows. The yellow line circle indicates that all J and T are associated with TJ, the dotted blued lined circle that all the Uk are associated with KU, and the black line circle that the mtDNA haplotype others are related in part to HV (dotted arrow) and the C-others (bold arrow). In **b**) the frequency in the studied population of the mtDNA haplogroups and **c**) the frequency of the mtDNA haplogroup clusters; the column indicates the frequency for all the population; NP, the number of no-progressors; P, the number of the progressors. Progressors and no-progressors are defined in the Methods section.

**Additional file 3.** Data of the frequency of single nucleotide polymorphism (SNP) genes and association of the mtDNA haplogroups with the clusters and their frequencies in the studied population (901).

**Additional file 4. **The optimum values of the different machine learning tools. DT, Decision Tree; ELM, Extreme Learning Machine; SA-ELM, Self-Adaptive Extreme Learning Machine; DT-SA-ELM, Decision Tree and Self-Adaptive Extreme Learning Machine; KNN, K-Nearest Neighbor; RF, Random Forest; SVM, Support Vector Machine; L1QP, L1 soft-margin minimization by quadratic programming; SMO, Sequential Minimal Optimization.

**Additional file 5.** Methods of the implementation of the machine learning classification methodologies.

**Additional file 6: Figure S3.** K-fold cross-validation methodology. All individuals were randomly divided equally into ten different groups. One group was reserved as a test sample (validation), and the nine remaining groups were considered as training (train) samples.

**Additional file 7: Figure S4.** Ten-fold cross-validation of the M32-3 and MH17 models. Validation of the model **a**) M32-3 (age, bone mass index [BMI], *TP63*, *DUS4L*, *GDF5*, *FTO*) and **b**) MH17 (age, BMI, mtDNA haplogroup, *FTO*, *SUPT3H*) was done using the ten-fold cross-validation (k-fold) methodology, as described in the Methods section and Additional file 4. Train, training stage; test, testing stage.

## Data Availability

Data from the Osteoarthritis Initiative (OAI) cohort used in this study is publicly available (https://data-archive.nimh.nih.gov/oai/). Data regarding the mtDNA haplogroups/clusters are part of the public OAI database. The nuclear SNP data have been submitted to the OAI and are at present blinded. The additional data used and analyzed for the current study are available from the corresponding author upon reasonable request, as long as the request is evaluated as scientifically relevant.

## Abbreviations

APOE4: *Apolipoprotein E* Gene
BMI: Body mass index
DMOAD: Disease-modifying osteoarthritis drug
DNA: Deoxyribonucleic acid
DT: Decision tree
DT-SA-ELM: Decision tree and self-adaptive ELM
DT-SAELM: Self-adaptive ELM
*DUS4L*: Dihydrouridine synthase 4 like
ELM: Extreme learning machine
*FTO*: FTO alpha-ketoglutarate-dependent dioxygenase
*GDF5*: Growth differentiation factor 5
*GNL3*: G protein nucleolar 3
GWAS: Genome-side association
JSN: Joint space narrowing
JSW: Joint space width
KL: Kellgren-Lawrence
KNN: K-nearest neighbor
M: Model
*MCF2L*: MCF.2 cell line derived transforming sequence like
ML: Machine learning
MRI: Magnetic resonance imaging
mtDNA: Mitochondrial DNA
No.: Number
OA: Osteoarthritis
OAI: Osteoarthritis Initiative
PCR: Polymerase chain reaction
PVBSP: Probability values of being progressors
RF: Random forest
SA-ELM: Self-adaptive extreme learning machine
SBE: Single base extension
SNP: Single nucleotide polymorphism
*SUPT3H*: SPT3 homolog
SVM: Support vector machine
TASOAC: Tasmanian Older Adult Cohort Study
*TGFA*: Transforming growth factor alpha
*TP63*: Tumor protein P63
WOMAC: Western Ontario and McMaster Universities Osteoarthritis Index
