# Supplementary material for: Single nucleotide polymorphism genes and mitochondrial DNA haplogroups as biomarkers for early prediction of knee osteoarthritis structural progressors: use of supervised machine learning classifiers
Source: BMC Med. 2022 Sep 12;20:316. doi: 10.1186/s12916-022-02491-1 (PMC9465912; doi:10.1186/s12916-022-02491-1)
Supplement: Supplementary file 1 — Additional file 1: Fig. S1. Frequency of single nucleotide polymorphism (SNP) genes in the studied population (n=901). We performed a dominant model of the risk alleles for the 8 SNPs: rs12107036.TP63, rs4730250. DUS4L, rs10948172.SUPT3H, rs11842874.MCF2L, rs8044769.FTO, rs11177.GNL3, rs143383.GDF5, rs3771501.TGFA. The column indicates the frequency for all the population. No, absence of the allele; NP, the number of no-progressors; P, the number of progressors; Yes, presence of the allele. Progressors and no-progressors are defined in the Methods section. [file 12916_2022_2491_MOESM1_ESM.docx]

**Additional file 1: Figure S1. Frequency of single nucleotide polymorphism (SNP) genes in the studied population (n=901)**

**
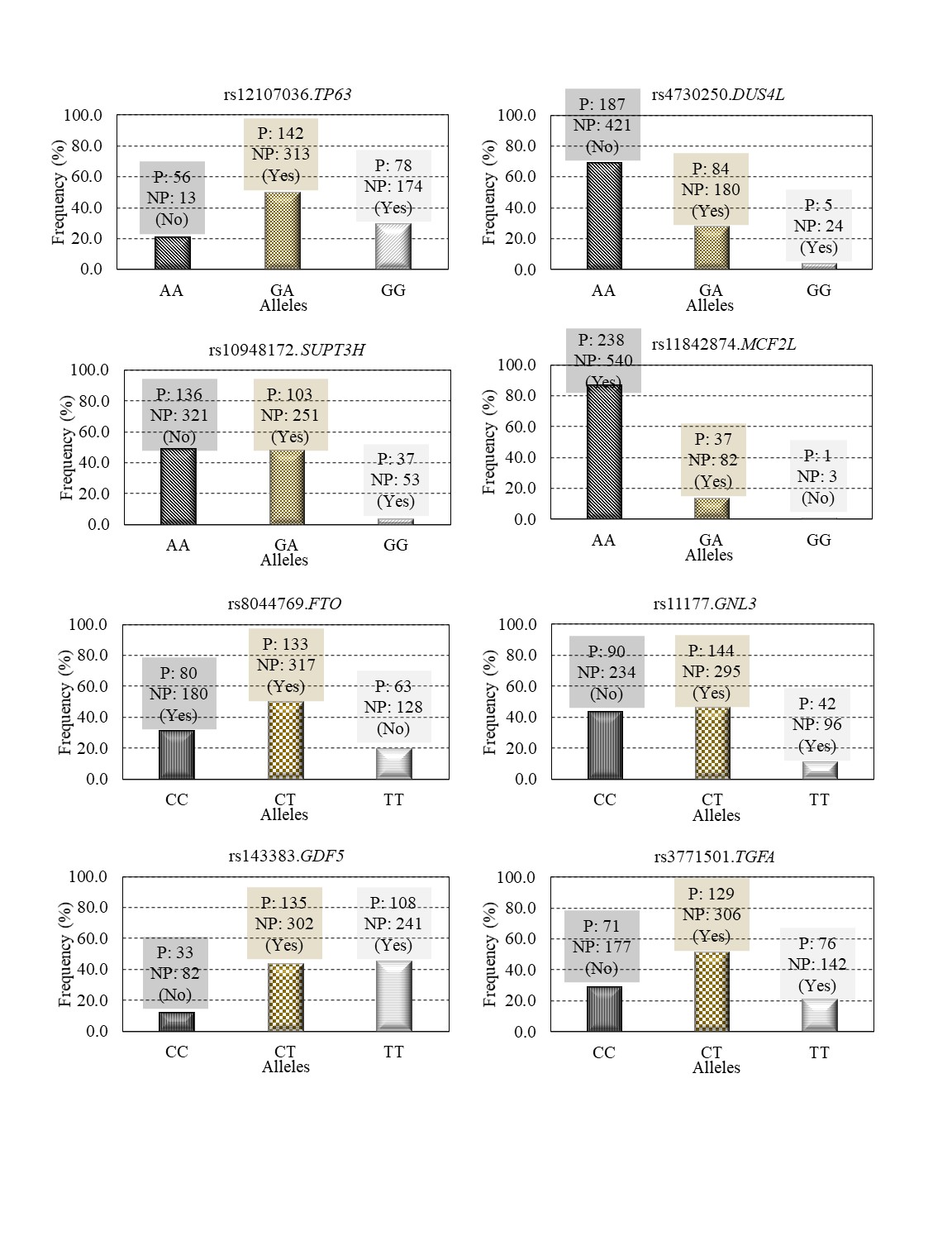
**

We performed a dominant model of the risk alleles for the 8 SNPs: rs12107036.*TP63*, rs4730250. *DUS4L*, rs10948172.*SUPT3H*, rs11842874.*MCF2L*, rs8044769.*FTO*, rs11177.*GNL3*, rs143383.*GDF5*, rs3771501.*TGFA*. The column indicates the frequency for all the population. No, absence of the allele; NP, the number of no-progressors; P, the number of progressors; Yes, presence of the allele. Progressors and no-progressors are defined in the Methods section.
