# Supplementary material for: Single nucleotide polymorphism genes and mitochondrial DNA haplogroups as biomarkers for early prediction of knee osteoarthritis structural progressors: use of supervised machine learning classifiers
Source: BMC Med. 2022 Sep 12;20:316. doi: 10.1186/s12916-022-02491-1 (PMC9465912; doi:10.1186/s12916-022-02491-1)
Supplement: Supplementary file 2 — Additional file 2: Fig. S2. Association and frequency of mtDNA haplogroups and clusters in the studied population (n=901). a) Association between mtDNA haplogroups and clusters. The number of participants is indicated in parenthesis and above the arrows. The yellow line circle indicates that all J and T are associated with TJ, the dotted blued lined circle that all the Uk are associated with KU, and the black line circle that the mtDNA haplotype others are related in part to HV (dotted arrow) and the C-others (bold arrow). In b) the frequency in the studied population of the mtDNA haplogroups and c) the frequency of the mtDNA haplogroup clusters; the column indicates the frequency for all the population; NP, the number of no-progressors; P, the number of the progressors. Progressors and no-progressors are defined in the Methods section. [file 12916_2022_2491_MOESM2_ESM.docx]

**Additional file 2: Figure S2. Association and frequency of mtDNA** **haplogroups and clusters in the studied population (n=901)**

**
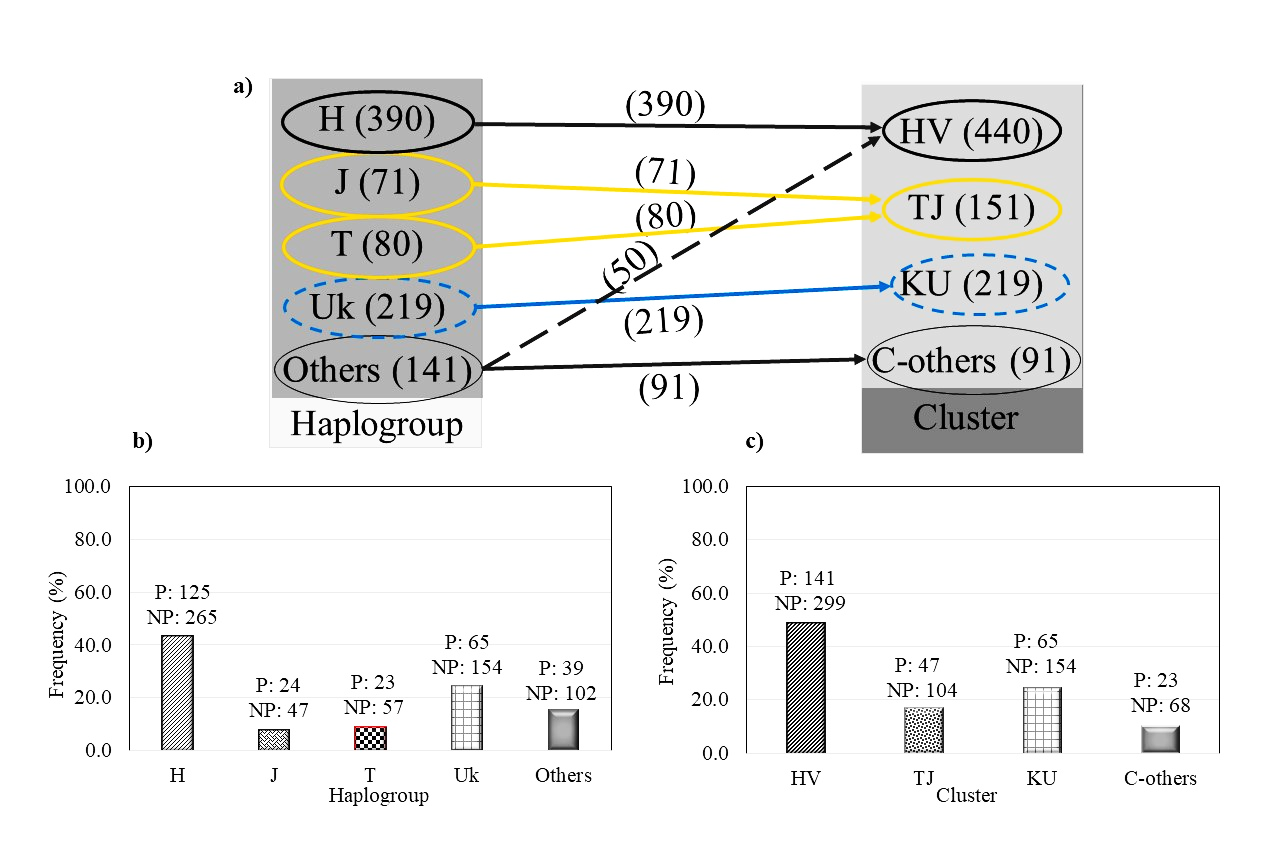
**

**a)** Association between mtDNA haplogroups and clusters. The number of participants is indicated in parenthesis and above the arrows. The yellow line circle indicates that all J and T are associated with TJ, the dotted blued lined circle that all the Uk are associated with KU, and the black line circle that the mtDNA haplotype others are related in part to HV (dotted arrow) and the C-others (bold arrow). In **b)** the frequency in the studied population of the mtDNA haplogroups and **c)** the frequency of the mtDNA haplogroup clusters; the column indicates the frequency for all the population; NP, the number of no-progressors; P, the number of the progressors. Progressors and no-progressors are defined in the Methods section.
