# Supplementary material for: Single nucleotide polymorphism genes and mitochondrial DNA haplogroups as biomarkers for early prediction of knee osteoarthritis structural progressors: use of supervised machine learning classifiers
Source: BMC Med. 2022 Sep 12;20:316. doi: 10.1186/s12916-022-02491-1 (PMC9465912; doi:10.1186/s12916-022-02491-1)
Supplement: Supplementary file 3 — Additional file 3. Data of the frequency of single nucleotide polymorphism (SNP) genes and association of the mtDNA haplogroups with the clusters and their frequencies in the studied population (901). [file 12916_2022_2491_MOESM3_ESM.docx]

**Additional file 3. Data**

**Frequency of single nucleotide polymorphism (SNP) genes in the studied population (n=901)**

We performed a dominant model of the risk alleles for the following eight SNPs: rs12107036 at *TP63*, rs8044769 at *FTO*, rs11177 at *GNL3*, rs4730250 at *DUS4L*, rs143383 at *GDF5*, rs10948172 at *SUPT3H*, rs11842874 at *MCF2L*, and rs3771501 at *TFGA*.

In the additional file 1, Figure S1, the three main genotypes (absence of the alleles AA, presence of GA and GG) of rs12107036 at *TP63* showed a percentage for all individuals (both progressors and no-progressors) of 21.5%, 50.5%, and 28.0%, respectively. The highest percentage was related to GA, and GG and AA were ranked second and third, respectively. For progressor individuals, the rank of all features was similar for all individuals, while for no-progressor individuals, the rank of GG and AA was changed in that GA, AA, and GG were ranked first to third, respectively.

For rs4730250 at *DUS4L* and rs11842874 at *MCF2L*, the alleles AA had the highest frequency (67.5% and 86.4%, respectively) for all individuals, GA (29.3% and 13.2%) ranked second, and GG (3.2% and 0.4%) was very low and ranked third. These ranks were also true when divided into progressors and no-progressors.

The percentages of rs10948172 at *SUPT3H* alleles AA (50.7%) and GA (39.3%) for all individuals were close, while GG (10%) ranked third. As for *DUS4L* and *MCF2L*, these ranks applied for progressors or no-progressors.

The rs8044769 at *FTO*, the alleles CT had the highest percentage for all groups (about 50.0%), CC was second (29.0%) and close to TT (about 21.5%). The rank for *GNL3* genotypes was similar to *FTO*.

For rs143383 at *GDF5*, the CT alleles were the highest (49.0% for each group), TT second (39.0%), and CC third (about 12.6%). The percentages of each feature in all, progressor and no-progressor individuals, were very similar.

The frequency distribution of all alleles for rs3771501 at *TFGA* was relatively similar to those of *FTO*, CT had the highest percentage, and CC and TT were ranked second and third, respectively.

**Association of the mtDNA haplogroups with the clusters and their frequencies in the studied population (n=901)**

Additional file 2, Figure S2a presents the association of mtDNA haplogroups and clusters. Data indicated that the cluster of HV is associated with 100% of H and 34.5% of the other mtDNA haplogroups. The total individuals of the T and J mtDNA haplogroups are associated with TJ. The cluster KU is associated with the total individuals of Uk. Finally, the cluster C-others is associated with 64.5% of the other mtDNA haplogroups.

In Additional file 2, Figure S2b, the percentage frequency of each haplotype was 43.3%, 7.9%, 8.9%, 24.3%, and 15.7%, for H, J, T, Uk, and others, respectively. As expected (1), the highest percentage was related to the haplotype H. The percentages of the haplotypes Uk, others, T, and J were ranked second to fifth, respectively. Comparison between the progressors and no-progressors showed that each mtDNA haplogroup had a very similar percentage. For the progressors, the mtDNA haplogroups H, J, T, Uk, and others are found in 45.3%, 8.7%, 8.3%, 23.6%, and 14.1%, respectively of the studied population, and in 42.4%, 7.5%, 9.1%, 24.7%, and 16.4% of no-progressors.

For the clusters (Additional file 2, Figure S2c), the percentage of HV, TJ, KU, and C-others for all individuals (progressors and no-progressors) was 48.8%, 16.8%, 24.3%, and 10.1%, respectively, in which the highest percentage was related to HV. The haplotype clusters KU, TJ, and C-others were ranked second to fourth, respectively. As for the haplotypes, the percentage of each cluster was very similar between progressors (HV, TJ, KU, and C-others: 51.1%, 17.0%, 23.6% and 8.3%) and no-progressors (47.8%, 16.6%, 24.6%, and 10.9%).

**Reference**

1. Martinez-Redondo D, Marcuello A, Casajus JA, Ara I, Dahmani Y, Montoya J, et al. Human mitochondrial haplogroup H: the highest VO2max consumer--is it a paradox? Mitochondrion. 2010;10(2):102-7.
