# Supplementary material for: Single nucleotide polymorphism genes and mitochondrial DNA haplogroups as biomarkers for early prediction of knee osteoarthritis structural progressors: use of supervised machine learning classifiers
Source: BMC Med. 2022 Sep 12;20:316. doi: 10.1186/s12916-022-02491-1 (PMC9465912; doi:10.1186/s12916-022-02491-1)
Supplement: Supplementary file 4 — Additional file 4. The optimum values of the different machine learning tools. DT, Decision Tree; ELM, Extreme Learning Machine; SA-ELM, Self-Adaptive Extreme Learning Machine; DT-SA-ELM, Decision Tree and Self-Adaptive Extreme Learning Machine; KNN, K-Nearest Neighbor; RF, Random Forest; SVM, Support Vector Machine; L1QP, L1 soft-margin minimization by quadratic programming; SMO, Sequential Minimal Optimization. [file 12916_2022_2491_MOESM4_ESM.docx]

**Additional file 4. The optimum values of the different machine learning tools**

| **Method** | **Parameter** | | **Setting** |
| --- | --- | --- | --- |
| **DT** | MinParent (Minimum number of branch node observations) | Men | 31 |
|  |  | Women | 50 |
| **ELM & SA-ELM** | Activation function | | hyperbolic tangent |
|  | Number of hidden neurons | Men | 19 |
|  |  | Women | 30 |
| **DT-SA-ELM** | MinParent | Men | 31 |
|  |  | Women | 50 |
|  | Activation function | | hyperbolic tangent |
|  | Number of hidden neurons | Men | 17 |
|  |  | Women | 29 |
| **KNN** | Minimum parent size | Men | 8 |
|  |  | Women | 4 |
|  | Distance | | cityblock |
|  | DistanceWeight | | equal |
|  | Standardize | | off |
| **SVM** | Kernel Function | | gaussian |
|  | Solver | | L1QP/SMO |
|  | ScoreTransform | | ismax |
|  | Box constraint | | 1 |
|  | KernelScale | | 1 |
|  | Standardize | | off |
|  | Alpha | | 0 |
|  | CacheSize | | 1000 |
|  | Verbose | | 0 |
|  | Prior probabilities | | empirical |
|  | IterationLimit | | 1.00E+06 |
| **RF** | Seed | | 100 |
|  | mtry (number of predictors considered at each split) | | 3 |
|  | ntree (number of trees in the forest) | | 5 |

DT, Decision Tree; ELM, Extreme Learning Machine; SA-ELM, Self-Adaptive Extreme Learning Machine; DT-SA-ELM, Decision Tree and Self-Adaptive Extreme Learning Machine; KNN, K-Nearest Neighbor; RF, Random Forest; SVM, Support Vector Machine; L1QP, L1 soft-margin minimization by quadratic programming; SMO, Sequential Minimal Optimization.
