# Supplementary material for: Single nucleotide polymorphism genes and mitochondrial DNA haplogroups as biomarkers for early prediction of knee osteoarthritis structural progressors: use of supervised machine learning classifiers
Source: BMC Med. 2022 Sep 12;20:316. doi: 10.1186/s12916-022-02491-1 (PMC9465912; doi:10.1186/s12916-022-02491-1)
Supplement: Supplementary file 5 — Additional file 5. Methods of the implementation of the machine learning classification methodologies. [file 12916_2022_2491_MOESM5_ESM.docx]

**Additional file 5. Methods**

**Implementation of the machine learning classification methodologies**

The random forest (RF) was implemented using the R package “RandomForest” (1), while the others were developed in the MATLAB environment. K-Nearest Neighbor (KNN), support vector machine (SVM), and decision tree (DT) were implemented using the “fitcknn”, “[fitcsvm](file:///C:\\Program%20Files\\MATLAB\\R2017b\\help\\stats\\fitcsvm.html?searchHighlight=fitcsvm&searchResultIndex=1)”, and “fitctree” function, respectively. Extreme learning machine (ELM) and self-adaptive ELM (SA-ELM) were codes developed by Huang et al. (2) and Cao et al. (3), respectively. Moreover, the combination of decision tree and self-adaptive ELM (DT-SA-ELM) is a combination of DT provided in the “fitctree” function in MATLAB and SA-ELM provided by Cao et al. (3). The optimum parameters and settings of all the above-mentioned ML tools in probability values of being structural progressor (PVBSP) forecasting, found through the trial-and-error process, are provided in Additional file 4.

**References**

1. Liaw A, Wiener M. Classification and regression by random forest. R News. 2002;2/3:18-22.

2. Huang GB, Zhu QY, Siew CK, editors. Extreme learning machine: a new learning scheme of feedforward neural networks. International Joint Conference on Neural Networks (IJCNN 2004); 2004 25-29 July; Budapest, Hungary.

3. Cao J, Lin Z, Huang G-B. Self-Adaptive Evolutionary Extreme Learning Machine. Neural Processing Letters. 2012;36.
