# Supplementary material for: Single nucleotide polymorphism genes and mitochondrial DNA haplogroups as biomarkers for early prediction of knee osteoarthritis structural progressors: use of supervised machine learning classifiers
Source: BMC Med. 2022 Sep 12;20:316. doi: 10.1186/s12916-022-02491-1 (PMC9465912; doi:10.1186/s12916-022-02491-1)
Supplement: Supplementary file 6 — Additional file 6: Figure S3. K-fold cross-validation methodology. All individuals were randomly divided equally into ten different groups. One group was reserved as a test sample (validation), and the nine remaining groups were considered as training (train) samples. [file 12916_2022_2491_MOESM6_ESM.docx]

**Additional file 6: Figure S3. K-fold cross-validation methodology**

**
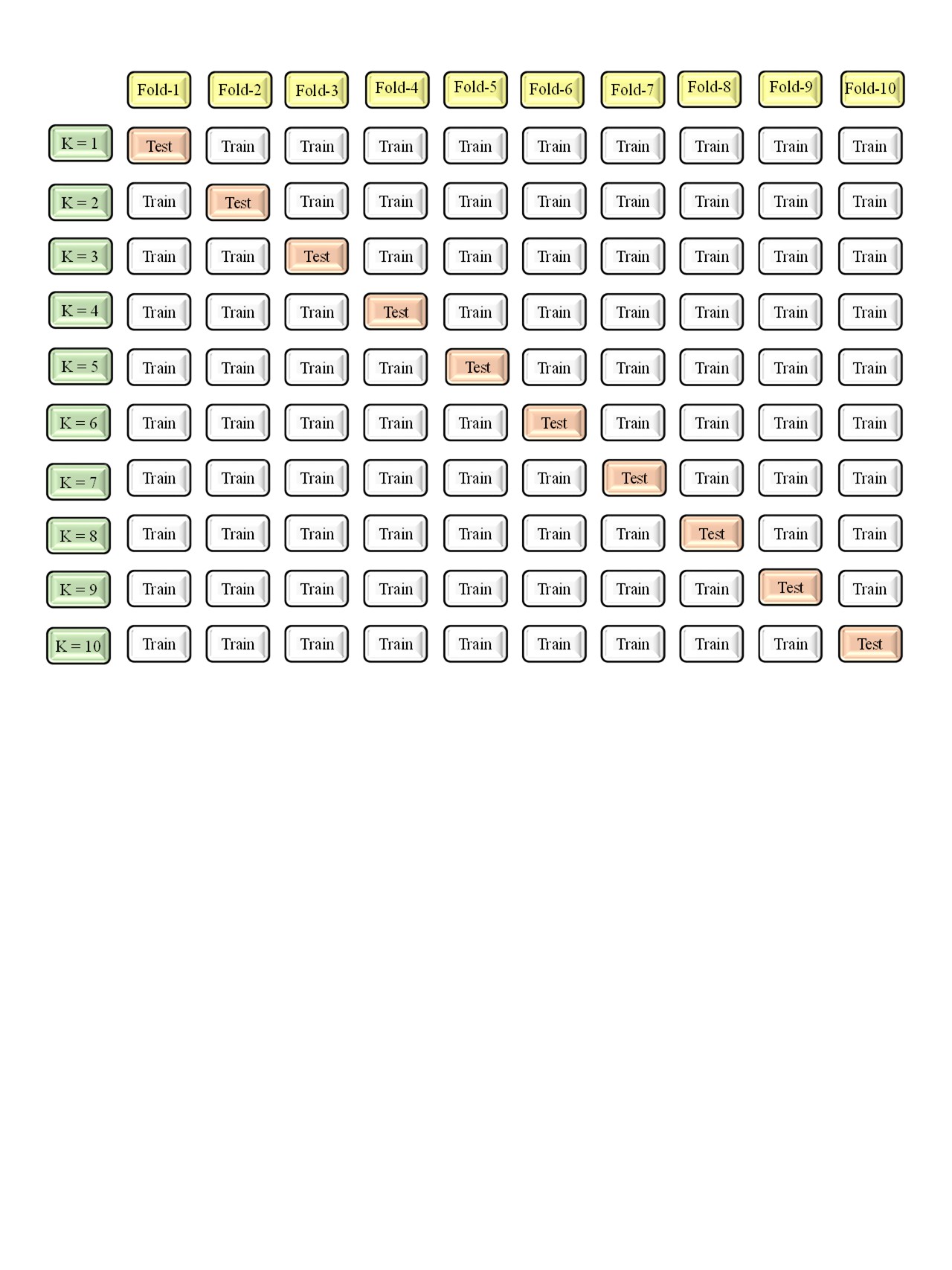
**

All individuals were randomly divided equally into ten different groups. One group was reserved as a test sample (validation), and the nine remaining groups were considered as training (train) samples.
