# Supplementary material for: Single nucleotide polymorphism genes and mitochondrial DNA haplogroups as biomarkers for early prediction of knee osteoarthritis structural progressors: use of supervised machine learning classifiers
Source: BMC Med. 2022 Sep 12;20:316. doi: 10.1186/s12916-022-02491-1 (PMC9465912; doi:10.1186/s12916-022-02491-1)
Supplement: Supplementary file 7 — Additional file 7: Figure S4. Ten-fold cross-validation of the M32-3 and MH17 models. Validation of the model a) M32-3 (age, bone mass index [BMI], TP63, DUS4L, GDF5, FTO) and b) MH17 (age, BMI, mtDNA haplogroup, FTO, SUPT3H) was done using the ten-fold cross-validation (k-fold) methodology, as described in the Methods section and Additional file 4. Train, training stage; test, testing stage. [file 12916_2022_2491_MOESM7_ESM.docx]

**Additional file 7: Figure S4. Ten-fold cross-validation of the M32-3 and MH17 models**


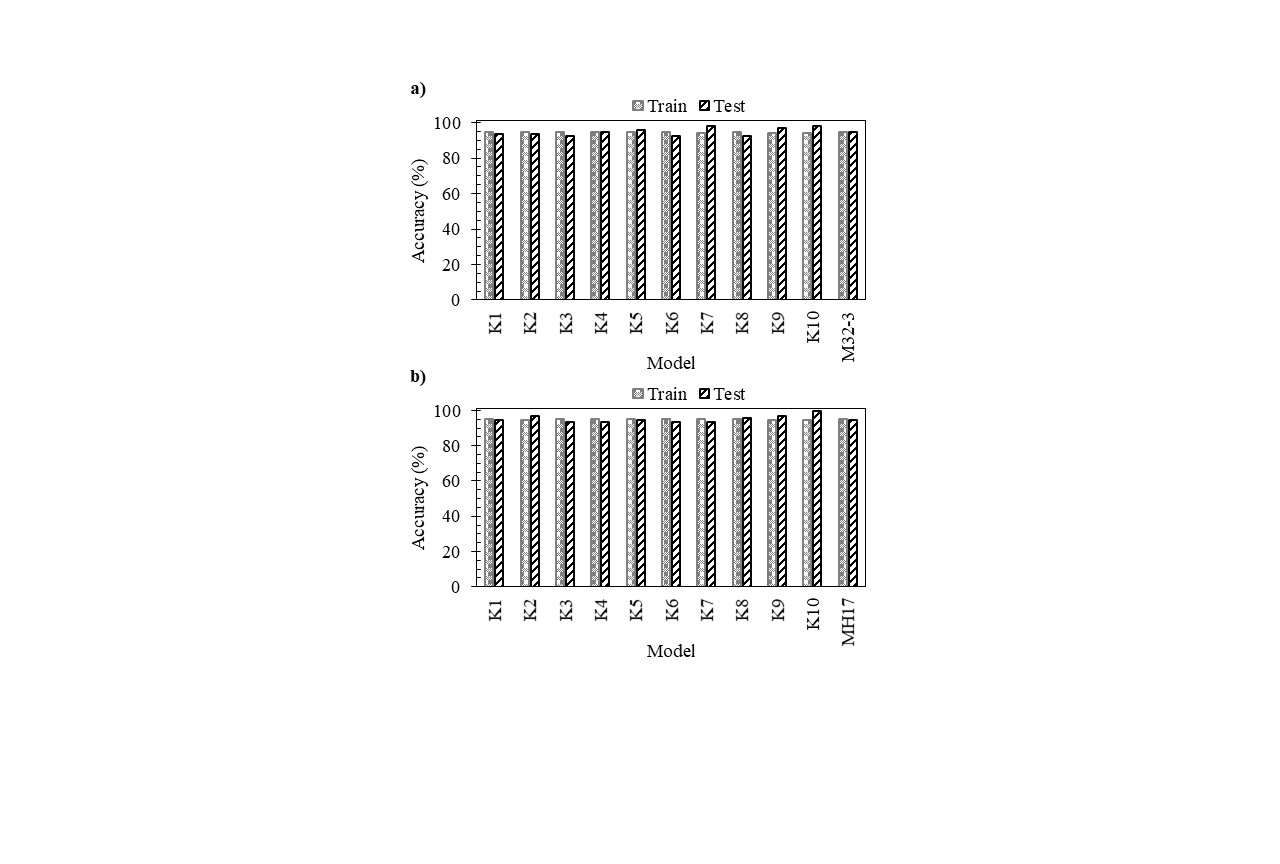


Validation of the model **a)** M32-3 (age, bone mass index [BMI], *TP63*, *DUS4L*, *GDF5*, *FTO*) and **b)** MH17 (age, BMI, mtDNA haplogroup, *FTO*, *SUPT3H*) was done using the ten-fold cross-validation (k-fold) methodology, as described in the Methods section and Additional file 6. Train, training stage; test, testing stage.
